# Antihyaluronidase and Alkaline Phosphatase (ALP) Activities of Medicinal Plants to Combat *Echis carinatus* Venom-Induced Toxicities

**DOI:** 10.1155/2021/6618349

**Published:** 2021-03-16

**Authors:** Syeda Fatima, Nazia Aslam, Sofia Khalid, Kalim Ullah, Khizar Abbas, Shahzad Hussain, Syed Sajid Hussain Shah, Zia-Ur-Rahman Qureshi, Mughal Qayum, Muhammad Hassham Hassan Bin Asad

**Affiliations:** ^1^Department of Environmental Sciences, Fatima Jinnah Women University, Rawalpindi, Pakistan; ^2^Department of Zoology, Kohat University of Science and Technology (KUST), Kohat, Pakistan; ^3^Department of Pharmacognosy, Faculty of Pharmacy, B.Z. University, Multan, Pakistan; ^4^Drugs Control & Traditional Medicines Division, National Institute of Health, Islamabad, Pakistan; ^5^Department of Environmental Sciences, COMSATS University Islamabad, Abbottabad Campus, 22060 KPK, Pakistan; ^6^Department of Pharmacy, SBK Women University, Quetta, Baluchistan, Pakistan; ^7^Department of Pharmacy, Kohat University of Science and Technology (KUST), Kohat, Pakistan; ^8^Department of Pharmacy, COMSATS University Islamabad, Abbottabad Campus, 22060 KPK, Pakistan

## Abstract

Snakebite is one of the most neglected diseases of developing countries. Deaths due to snakebite envenoming are quite high in Pakistan, and many deaths are caused by *Echis carinatus* envenomation. Traditional use of medicinal plants against snakebites is a common practice in Pakistan due to countless benefits. The current study was performed with the objective to evaluate eighteen Pakistani medicinal plants inhibitory potential against hyaluronidase and alkaline phosphatase enzymes of Pakistani *Echis carinatus* venom. Hyaluronidase activity (0.2-1.6 mg/0.1 mL) and alkaline phosphatase activity (0.1-0.8 mg/0.1 mL) were measured in dose-dependent manner. Crude methanolic extracts of medicinal plants were used for in vitro investigation of their inhibitory activity against toxic enzymes. All active plants were fractioned using different solvents and were again analyzed for inhibitory activity of same enzymes. Results indicated all plants were able to neutralize hyaluronidase that *Swertia chirayita* (Roxb. ex Flem.) Karst., *Terminalia arjuna* Wight and Arn, *Rubia cordifolia* Thumb., and *Matthiola incana* (L.) R.Br. inhibited maximum hyaluronidase activity equivalent to standard reference (*p* > 0.5). Pakistani medicinal plants are dense with natural neutralizing metabolites and other active phytochemicals which could inhibit hyaluronidase activity of Pakistani *Echis carinatus* venom. Further advanced studies at molecular level could lead us to an alternative for envenoming of Pakistani *Echis carinatus* venom.

## 1. Introduction

Venomous snakes are among the most feared animals on planet earth [1]. Snakebite is a common public health problem worldwide which not only cause disabilities is the victims but also results in huge number of deaths annually [2]. According to the World Health Organization (WHO), snakebite injury has been declared as “disease of poverty” as it is observed to effect mostly in rural communities of third world countries [3]. Epidemiological data showed that over 2.5 million snakebites occur annually resulting in 125,000 deaths [4–6]. Pakistan is among the highest snakebite-affected countries of Asia with 40,000 envenoming and 8,200 deaths annually [7]. Venomous snakes have been grouped among four major families containing more than 200 venomous snake species worldwide [8]. Snakebite envenoming results in minor as well as major consequences depending on the venom of particular snake species. Effects of envenoming include pain, edema, hypotension, necrosis, cardiac arrest, paralysis, mucus discharge, bleeding gums, bleeding wounds, hematuria, and eventually death [9, 10].

Venomous snakes of Pakistan are mostly from Elapidae and Viperdae family [11]. One of the most toxic viper *Echis carinatus*, also known as saw-scaled viper, is found in Ashtola Island of Makran (Baluchistan) and deserts of Thar (Sindh) and Cholistan (Punjab), Pakistan. *Echis carinatus* snake has around 0.6 m length, flat body, pointed tail, and is known as a true viper [7, 12]. *Echis carinatus* envenoming effects anticoagulant or procoagulant activity due to presence of active enzymes in its venom which in turn disturbs the hemostatic system [13, 14]. Envenomation of Echis also cause local tissue damage and cell necrosis by the synergistic effect of hydrolytic enzymes hyaluronidases, phospholipases *A*_2_, and proteases [10]. Hyaluronidase enzymes of snake venom are known as spreading factors as they attack on glycosidic linkage of hyaluronic acid and release small sugar molecules such as chondroitin and chondroitin sulfates which result in destruction of extracellular matrix [15]. Alkaline phosphatases (ALPases) are very poisonous enzymes as they can nonspecifically hydrolyze phosphate esters [16]. ALPase produces adenosine which induces miscellaneous hazardous effects like hypertention, cardiotoxitiy, redness, inflammation, antiplatelet aggregation, renal failure, unconsciousness, pain, and analgesia [17].

An estimation by WHO states that more than 80% of the world population rely on herbal-based traditional medicines for treatment of different ailments [18]. Medicinal plants have vital role in modern age drugs and folk medicines as they have various metabolites having antimicrobial, antiasthmatic, antiallergic, antidiabetic, and antisnake venom properties. In recent few decades, screening of medicinal plant materials has been considered important due to its curative properties against numerous diseases including snakebite. Nowadays, attempts are being made by different health investigators to produce a plant-based alternative instead of antiserum [19]. Pakistan has rich plant diversity, and most of rural communities depend on local medicinal flora for health-related issues. The present study is based on traditionally used indigenous medicinal plants inhibitory potential against hyaluronidases and alkaline phosphatase enzymes present in Pakistani *Echis carinatus* venom.

## 2. Materials and Methods

### 2.1. *Echis carinatus* Venom

*Echis carinatus* lyophilized venom was provided by the National Institute of Health, Islamabad, Pakistan. It was kept in sterilized light resistant bottle and was stored at 4-8°C. Venom concentration was used in terms of dry weight.

### 2.2. Chemical Reagents

All the chemicals for the present study were purchased from Merck and were of analytical grade.

### 2.3. Collection of Medicinal Plants

Medicinal plants selected for the current study were reported previously for therapeutic properties against snakebite. Plants were collected from different regions of Pakistan, whereas few of them were purchased from Pansara store, Naswari Baazar, Rawalpindi. After collection, plants were identified by expert botanist, and voucher specimen was deposited in herbarium of Institute of Pure and Applied Biology, BZU, Multan, Pakistan. List of medicinal plants is summarized in Table 1.

### 2.4. Plant Material Extraction

Shade dried plants (part) were chopped and subjected to simple maceration process. Methanol was used as solvent, and dried powder of desired part(s) of plant was soaked in the solvent. All soaked plants were kept at ambient temperature for about a month. Two-way filtration was done firstly by using normal filter paper and then with Whatman filter paper 41. After that, the solvent was evaporated to obtain extracts which were stored for further research [20].

### 2.5. Hyaluronidase Assay

The enzymatic assay of *Echis carinatus* hyaluronidase enzyme was performed by using method of Pukrittayakamee et al. [21] with slight modification. Briefly, the assay mixture contained acetate buffer (0.2 M sodium acetate–acetic acid, pH 5.0, containing 0.15 M NaCl), 50 *μ*g of hyaluronic acid (0.5 mg/mL in buffer), and enzymes in a final volume of 1.0 mL. The mixture was incubated for 15 minutes at 37°C, and the reaction was quenched by the addition of 2 mL of 2.5% CTAB in 2% NaOH. The absorbance was read at 400 nm (within ten minutes) against a control solution containing 1 mL of the same buffer and 2 mL of 2.5% CTAB in 2% NaOH. Percentage of the remaining hyaluronic acid was considered as turbidity-reducing activity taking absorbance of sample with no enzyme added as 100%. One unit was defined as the amount of enzyme that induced 50% turbidity reduction. Kinetics data to optimize the hyaluronidase assay were also obtained [22, 23]. To measure medicinal plants neutralizing potential, (0.8 mg) extracts were preincubated with venom for 15 min at 37°C.

### 2.6. Alkaline Phosphatase Assay

Reaction mixture was prepared by mixing 0.5 mL of 0.5 M glycine buffer (pH = 8.5), 0.5 mL of 0.01 M *p*-nitrophenyl phosphate, and 0.3 mL of 0.01 M MgSO_4_. Subsequently, venom (0.1-0.8 mg/0.1 mL) was added. Reaction mixture was incubated at 37°C for half an hour. At the end of this period, 2 mL of NaOH (0.2 M) solution was added and was further kept for 20 minutes at room temperature to halt the reaction and to confer stable yellow color to *p*-nitrophenol which absorbed maximally at 400 nm [37]. A standard curve of known concentrations of *p*-nitrophenol was constructed, and ALPase activity will be expressed as micromole of product released per minute [38]. To measure neutralizing potential of medicinal plants, plant extracts were preincubated with venom for 30 min at 37°C [31].

### 2.7. Fractionation of Active Medicinal Plants

All the active medicinal plants were fractioned by using four solvents: *n*-hexane, chloroform, dichloromethane, and ethyl acetate [39]. Fractions were evaporated, and the obtained fractions were again tested using the previously described assay method for hyaluronidases activity.

### 2.8. Phytochemical Screening

Phytochemical screening for secondary metabolites present in both the active medicinal plants and active plant fraction was performed according to standard procedures [40].

### 2.9. Statistical Analysis

All experimental data was presented as mean, whereas inhibition of enzyme activity was expressed in percentage by using Microsoft Excel, 2007. Student's *t-*test was used to compare the inhibition results to the standard reference with level of significance set at *p* > 0.5.

## 3. Results

**Figure 1:**
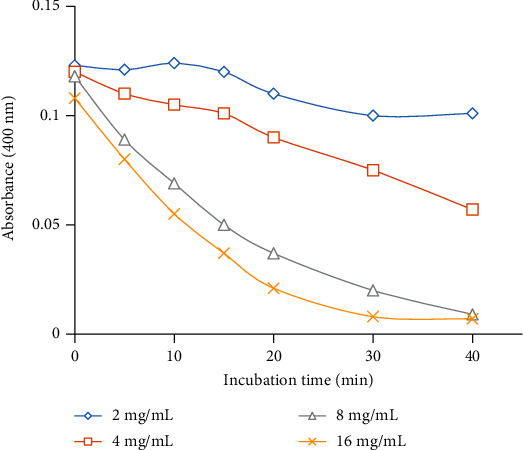
Effects of incubation time (37°C) on hyaluronidase activity at different venom concentrations of Pakistani *Echis carinatus* venom.

**Figure 2:**
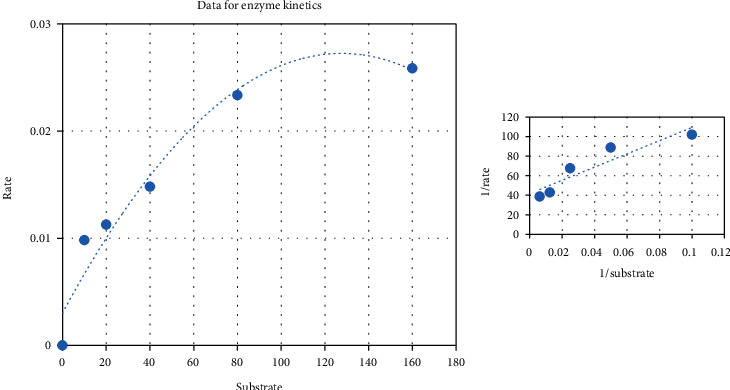
Michaelis-Menten kinetics data obtained for optimization of hyaluronidase enzyme of Pakistani *Echis carinatus* venom.

**Figure 3:**
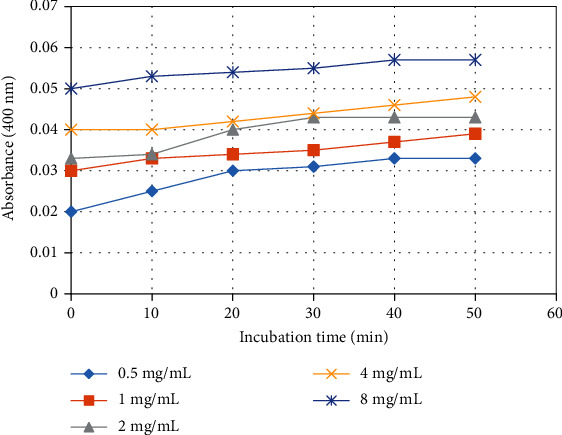
Screening of alkaline phosphatase enzymatic activity at different concentrations present in *Echis carinatus* venom.

Results showed that hyaluronidase enzyme (2-16 mg/mL) of *Echis carinatus* venom was found to reduce turbidity of reaction mixture upon incubating for longer duration of time. The decrease in turbidity was observed in dose-dependent manner (Figure 1). Venom at the higher concentration (8 mg/mL) was found to remove turbidity completely and considered to show 100% enzyme activity at 10 minutes. Furthermore, kinetics data (Michaelis-Menten kinetics) were also obtained (Km = 16; *V*_max_ = 0.023) to optimize the hyaluronidase assay (Figure 2). Results for fractionation of all active plants using four solvents showed *n*-hexane to be the most active fraction for all active plants, i.e., *Swertia chirayita* (Roxb. ex Flem.) Karst., *Terminalia arjuna* (DC) Wight and Arn, *Matthiloa incana* (L.) R.Br., and *Rubia cordifolia* Thumb. with inhibition percentages 89%, 88%, 84%, and 86%, respectively (Table 2). Details of fractionation have summarized in Table 3. Alkaline phosphatase activity was determined by measuring micromoles of *p*-nitrophenol (product) released per minute by the colorimetric assay. Despite of high *Echis carinatus* venom doses (1-8 mg/mL), very minute response (0.057) was observed for triplicates within fifty minute incubation (Figure 3). Due to very less amount of alkaline phosphatase even at high venom doses which was undetectable, therefore, this assay was not performed further and rejected.

Eighteen Pakistani medicinal plants were tested to evaluate inhibition potential against hyaluronidase enzyme of Pakistani *Echis carinatus* venom. Out of all plants, *Matthiloa incana* (L.) R.Br., *Rubia cordifolia* Thumb., *Swertia chirayita* (Roxb. ex Flem.) Karst., and *Terminalia arjuna* (DC) Wight and Arn inhibited 88% (*p* > 0.5) and 88% (*p* > 0.5) while *Matthiloa incana* (L.) R.Br. and *Rubia cordifolia* Thumb inhibited 86% (*p* > 0.5) and 87% (*p* > 0.5), respectively. Inhibition potential of all medicinal plants against hyaluronidase enzyme activity of *Echis carinatus* venom has been summarized in Table 2. Presence of phytochemicals in all active plants and their active fractions were analyzed by following standard protocols, and results have been summarized in Tables 4–6.

## 4. Discussion

Snakebite envenoming is a common issue in many developing countries, and mostly rural communities suffer its consequences. They use medicinal plants for pain relief. At present, the only ideal treatment for snakebite injury is the use of antisnake venom serum which is not easily available. It is costly and requires specific storage protocol. People with such injuries have to rush to the hospital for treatment which gives venom the time to cause neurotoxicity, hemorrhage, and local tissue damage that are not reversible in the victims. Also, antisnake venom serum has some side effects like anaphylactic reaction, serum sickness, and many other problems [12, 41]. It is therefore necessary to find plant-based antidote to neutralize snake venom toxic effects. Use of medicinal plants is still a common practice by traditional healers as it is easily available natural resource and provides instant aid against snake envenoming [42, 43].

*Echis carinatus* is one the four deadliest snakes found in Pakistan. Its venom consists of many hydrolytic enzymes which acts together to spread toxins into its victim. Hyaluronidase enzyme being one of the toxic material degrade local/connective tissues and proteins which promote the diffusion of venom toxins resulting in cell damage, inflammation, reduced membrane viscosity, pathogenesis, and impaired immune system [44]. Severity of cell degradation and necrosis also depends on amount of venom injected [45]. Extracellular matrix degradation is a continuous process in victim; therefore, antisnake venom serum could not inhibit local tissue damage completely [44].

Hyaluronidase activity was measured by its turbidity-reducing property, and its inhibition was evaluated by medicinal plants. *Terminalia arjuna* (DC) Wight and Arn (Combretaceae) and *Rubia cordifolia* Thumb. (Rubiaceae) neutralized hyaluronidase enzyme activity of *Echis carinatus* venom with *p* > 0.5. Similarly in previous studies, medicinal plants from Combretaceae and Rubiaceae family have been proved to inhibit hyaluronidase enzyme activity in *Naja nigricollis* [46]. *Swertia chirayita* (Roxb. ex Flem.) Karst. and *Matthiloa incana* (L.) R.Br. also showed antihyaluronidase activity (*p* > 0.5) which was possible as medicinal plants are packed with inhibitory compounds like alkaloids, antioxidants, flavonoids, glycosides, glycosaminoglycans, oligosaccharides, fatty acids, polysaccharides, terpenoids, and other compounds with therapeutic properties [47]. Previous researches have showed many plants with potential to inhibit snake venom hyaluronidase activity. These plants include *Mangifera indica*, *Andrographis paniculata*, *Carisssa spinarum*, and *Azima tetracantha Lam* used against *Daboia russellii*, *Naja naja*, *Bungarus caeruleus* ,and *Vipera russelli*, respectively [48–51].

Alkaline phosphatases along with other enzymes (like phospholipases and proteases) of snake venom assist in cell necrosis which lead to liberation of nucleic acids (DNA and RNA) and eventually release adenosine which cause detrimental damage to human body [30, 52]. Alkaline phosphatase enzyme is less studied, and thus their role is not specified. In current study, alkaline activity was very minute and unmeasurable in spite of high venom doses; therefore, this assay was dropped. It was supported by previous studies which elaborated that alkaline phosphatase enzymes have been found more in snakes of Elapidae and Crotalidae than in venom of Viperidae snakes [17, 53].

Janardhan et al. [50] reported that *n*-hexane extract of *Azima tetracantha* Lam. inhibited hylauronidase activity in *Vipera russelli* whereas ethyl acetate extract inhibited *Bungarus caeruleus* hyaluronidase enzyme activity. Similarly, in the current study, fractionation data showed that *n*-hexane fractions of all active plants inhibited hyaluronidase activity, whereas some of ethyl acetate fractions were also able to neutralize enzyme activity.

## 5. Conclusions

Present study on toxic enzymes of Pakistani *Echis carinatus* venom revealed that traditionally used medicinal plants have great potential to inhibit hyaluronidase activity due to combined action of phytochemicals. Neutralizing activity of medicinal plants could be studied further on animals and other in vitro pharmacological testings in order to develop a natural or synthetic antidote from indigenous medicinal plants against snakebite envenoming.

## Figures and Tables

**Table 1:** Description of selected indigenous medicinal plants having neutralizing potential against snakebite.

Sr. no.	Botanical names of selected medicinal plants	Family	Parts used	Voucher number	References
1	*Adiantum capillus-veneris* L.	Adiantaceae	Whole plants	R.R. Stewart F.W. Pak. 4(2)	[24]
2	*Albizia lebbeck* (L.) Benth.	Mimosaceae	Seeds	R.R. Stewart F.W. Pak. 381(9)	[24]
3	*Althaea officinalis* L.	Malvaceae	Roots	R.R. Stewart F.W. Pak. 477(6)	[25]
4	*Calotropis procera* W. T. Aiton	Asclepiadaceae	Flowers	R.R. Stewart F.W. Pak. 566(6)	[26]
5	*Citrullus colocynthis* (L.) Schrad.	Cucurbitaceae	Fruits	R.R. Stewart F.W. Pak. 702(10)	[24]
6	*Curcuma longa* L.	Zingiberaceae	Rhizome	R.R. Stewart F.W. Pak. 66(3)	[27]
7	*Eclipta prostrata* L.	Asteraceae	Whole plants	R.R. Stewart F.W. Pak. 743(5)	[28]
8	*Eugenia jambolana* (Willd. ex O. Berg)	Myrtaceae	Seeds	R.R. Stewart F.W. Pak. 504(2)	[29].
9	*Fagonia cretica* L.	Zygophyllaceae	Leaves and twigs	R.R. Stewart F.W. Pak. 433(2)	[30]
10	*Matthiloa incana* (L.) R.Br.	Brassicaceae	Seeds	R.R. Stewart F.W. Pak. 322(2)	[31]
11	*Momordica charantia* L.	Cucurbitaceae	Fruits	R.R. Stewart F.W. Pak. 706(1)	[32]
12	*Trichodesma indicum* (L.) R. Br.	Boraginaceae	Leaves	R.R. Stewart F.W. Pak. 604(3)	[33]
13	*Psoralea corylifolia* L.	Fabaceae	Seeds	R.R. Stewart F.W. Pak. 418(1)	[34]
14	*Rubia cordifolia* Thumb.	Rubiaceae	Roots	R.R. Stewart F.W. Pak. 689(4)	[24]
15	*Sapindus mukorossi* Gaertn.	Sapindaceae	Fruits	R.R. Stewart F.W. Pak. 463(3)	[24]
16	*Swertia chirayita* (Roxb. ex Flem.) Karst.	Gentianaceae	Stems	R.R. Stewart F.W. Pak. 561(4)	[35]
17	*Terminalia arjuna* (DC) Wight and Arn	Combretaceae	Bark	R.R. Stewart F.W. Pak. 502(4)	[31]
18	*Lepidium sativum* L.	Brassicaceae	Whole plants	R.R. Stewart F.W. Pak. 319(4)	[36]

**Table 2:** Inhibitory potentials of indigenous medicinal plants against hyaluronidase activity of Pakistani *Echis carinatus* venom.

Sr. No.	Name of material used	Absorbance	Percentage protection (%)	*p* value
1	*Echis carinatus*	0.110 ± 0.006	0^∗∗^	*p* < <0.001
2	Saline (control)	0.210 ± 0.005	100^∗^	*p* > 0.5
3	*Adiantum capillus-veneris* L.	0.165 ± 0.005	61^∗∗^	0.5 > *p* > 0.1
4	*Albizia lebbeck* (L.) Benth.	0.167 ± 0.001	63^∗∗^	0.5 > *p* > 0.1
5	*Althaea officinalis* L.	0.111 ± 0.001	1^∗∗^	*p* < <0.001
6	*Calotropis procera* W. T. Aiton	0.128 ± 0.05	20^∗∗^	0.1 > *p* > 0.02
7	*Citrullus colocynthis* (L.) Schrad.	0.129 ± 0.006	21^∗∗^	0.1 > *p* > 0.02
8	*Curcuma longa* L.	0.083 ± 0.001	-30^∗∗^	*p* < <0.001
9	*Eclipta prostrata* L.	0.171 ± 0.005	68^∗∗^	0.5 > *p* > 0.1
10	*Eugenia jambolana* (Willd. ex O. Berg)	0.088 ± 0.006	-24^∗∗^	*p* < <0.001
11	*Fagonia cretica* L.	0.174 ± 0.001	71^∗∗^	0.5 > *p* > 0.1
12	*Matthiola incana* (L.) R.Br.	0.187 ± 0.001	86^∗^	*p* > 0.5
13	*Momordica charantia* L.	0.132 ± 0.002	24^∗∗^	0.1 > *p* > 0.02
14	*Trichodesma indicum* (L.) R. Br.	0.119 ± 0.01	10^∗∗^	*p* < <0.001
15	*Psoralea corylifolia* L.	0.159 ± 0.02	54^∗∗^	0.5 > *p* > 0.1
16	*Rubia cordifolia* thumb.	0.188 ± 0.02	87^∗^	*p* > 0.5
17	*Sapindus mukorossi* Gaertn.	0.137 ± 0.002	30^∗∗^	0.5 > *p* > 0.1
18	*Swertia chirayita* (Roxb. ex Flem.) Karst.	0.190 ± 0.01	89^∗^	*p* > 0.5
19	*Terminalia arjuna* (DC) Wight and Arn	0.189 ± 0.03	88^∗^	*p* > 0.5
20	*Lepidium sativum* L.	0.155 ± 0.002	50^∗∗^	0.5 > *p* > 0.1
21	Rutin trihydrate (standard hyaluronidase inhibitor)	0.191 ± 0.01	90^∗∗∗^	Selected to compare

Note: ^∗^ represents *p* values nonsignificantly different from standard reference. ^∗∗^ represents *p* values significantly different from standard reference. ^∗∗∗^ represents value selected to compare.

**Table 3:** Fractionation of all active plants by different solvents inhibiting hyaluronidase enzyme activity of Pakistani *Echis carinatus* venom.

Botanical names	Percentage inhibition by different solvents (%)			
	*n*-hexane	Chloroform	Dichloromethane	Ethyl acetate
*Swertia chirayita* (Roxb. ex Flem.) Karst.	89	66	-88	83
*Terminalia arjuna* (DC) Wight and Arn	88	-61	23	85
*Rubia cordifolia* thumb.	87	64	83	51
*Matthiola incana* (L.) R.Br.	86	50	86	59

**Table 4:** Phytochemical analysis of active plant crude extracts evaluated as antivenom.

Phytochemicals	*Swertia chirayita* (Roxb. ex Flem.) Karst.	*Terminalia arjuna* (DC) Wight and Arn	*Rubia cordifolia* thumb.	*Matthiola incana* (L.) R.Br.
Alkaloids	**+**	**+**	**+**	**+**
Flavonoids	**+**	**+**	**+**	**+**
Fatty acids	-	-	-	-
Steroids	**+**	**+**	**+**	**+**
Phenols	**+**	**+**	**+**	**+**
Tannins	**+**	**+**	**+**	**+**
Glycosides	-	**+**	**+**	**+**
Saponins	**+**	**+**	**+**	**+**
Proteins	-	**+**	**+**	-
Terpenoids	-	**+**	**+**	**+**
Carbohydrate	-	**+**	-	**+**

Note: the + sign indicated about presence while the – sign depicted about absence.

**Table 5:** Phytochemical analysis all active fractions of *Swertia chirayita* (Roxb. ex Flem.) Karst. and *Terminalia arjuna* Wight and Arn extracts.

Phytochemicals	*Swertia chirayita* (Roxb. ex Flem.) Karst.		*Terminalia arjuna* (DC) Wight and Arn	
	*n*-hexane	Ethyl acetate	*n*-hexane	Ethyl acetate
Alkaloids	+	+	+	+
Flavonoids	+	+	+	+
Fatty acids	-	-	-	-
Steroids	+	+	+	+
Phenols	-	-	+	+
Tannins	+	-	+	+
Glycosides	+	+	-	+
Saponins	+	+	+	+
Proteins	-	-	-	-
Terpenoids	+	-	-	+
Carbohydrate	-	-	-	-

Note: the + sign indicated about presence while the – sign depicted about absence.

**Table 6:** Phytochemicals analysis all active fractions of *Rubia cordifolia* Thumb. and *Matthiola incana* (L.) R.Br. extracts.

Phytochemicals	*Rubia cordifolia* Thumb.		*Matthiola incana (*L.) R.Br.	
	*n*-hexane	Dichloromethane	*n*-hexane	Dichloromethane
Alkaloids	**+**	**+**	+	+
Flavonoids	**+**	-	+	+
Fatty acids	-	-	-	-
Steroids	**+**	**+**	+	+
Phenols	-	-	+	-
Tannins	-	-	+	+
Glycosides	-	-	+	+
Saponins	-	-	+	+
Proteins	-	**+**	-	+
Terpenoids	**+**	**+**	+	+
Carbohydrate	-	-	-	-

Note: the + sign indicated about presence while the – sign depicted about absence.

## Data Availability

Upon request data may be provided by the first author (Syeda Fatima: fatimaasyed90@gmail.com).
